# Extraction of Family History Information From Clinical Notes: Deep Learning and Heuristics Approach

**DOI:** 10.2196/22898

**Published:** 2020-12-29

**Authors:** João Figueira Silva, João Rafael Almeida, Sérgio Matos

**Affiliations:** 1 Department of Electronics, Telecommunications and Informatics Institute of Electronics and Informatics Engineering of Aveiro University of Aveiro Aveiro Portugal; 2 Department of Information and Communications Technologies University of A Coruña A Coruña Spain

**Keywords:** natural language processing, rule-based, deep learning, contextual embeddings, word embeddings, family medical history, information extraction, clinical notes, electronic health record

## Abstract

**Background:**

Electronic health records store large amounts of patient clinical data. Despite efforts to structure patient data, clinical notes containing rich patient information remain stored as free text, greatly limiting its exploitation. This includes family history, which is highly relevant for applications such as diagnosis and prognosis.

**Objective:**

This study aims to develop automatic strategies for annotating family history information in clinical notes, focusing not only on the extraction of relevant entities such as family members and disease mentions but also on the extraction of relations between the identified entities.

**Methods:**

This study extends a previous contribution for the 2019 track on family history extraction from national natural language processing clinical challenges by improving a previously developed rule-based engine, using deep learning (DL) approaches for the extraction of entities from clinical notes, and combining both approaches in a hybrid end-to-end system capable of successfully extracting family member and observation entities and the relations between those entities. Furthermore, this study analyzes the impact of factors such as the use of external resources and different types of embeddings in the performance of DL models.

**Results:**

The approaches developed were evaluated in a first task regarding entity extraction and in a second task concerning relation extraction. The proposed DL approach improved observation extraction, obtaining F_1_ scores of 0.8688 and 0.7907 in the training and test sets, respectively. However, DL approaches have limitations in the extraction of family members. The rule-based engine was adjusted to have higher generalizing capability and achieved family member extraction F_1_ scores of 0.8823 and 0.8092 in the training and test sets, respectively. The resulting hybrid system obtained F_1_ scores of 0.8743 and 0.7979 in the training and test sets, respectively. For the second task, the original evaluator was adjusted to perform a more exact evaluation than the original one, and the hybrid system obtained F_1_ scores of 0.6480 and 0.5082 in the training and test sets, respectively.

**Conclusions:**

We evaluated the impact of several factors on the performance of DL models, and we present an end-to-end system for extracting family history information from clinical notes, which can help in the structuring and reuse of this type of information. The final hybrid solution is provided in a publicly available code repository.

## Introduction

### Background

For many years, the rapid progress in technology has continually pushed the field of medicine forward, striving for the improvement of health care quality. Novel tools provide new possibilities, such as access to new types of information (eg, medical imaging and genome sequencing) and larger amounts of data, along with associated challenges such as how to store and organize the resulting vast amounts of multimodal medical information. The electronic health record (EHR) solves this by providing an electronic infrastructure for storing structured and unstructured information generated throughout time [1], thus maintaining the patient trajectories by maintaining a longitudinal view over the medical history of patients. Such data can then be explored for applications such as cohort selection [2] or to provide medical entities with clinical decision support [3-5].

Despite being harder to explore, unstructured data can contain relevant information that is not obtainable elsewhere [6], which is particularly evident in clinical notes, where medical narratives allow for more accurate and complete descriptions of medical situations [7]. As there is significant interest in exploring and reusing information from clinical notes, a possible approach is to process free text and extract relevant information that can be stored as structured data [7]. This process has historically been manual, consisting of having clinical experts review clinical notes in search for relevant information. However, heavy reliance on a manual component greatly limits the potential and usability of this process as it cannot scale with the increasing volumes of information [5].

Another possible solution for these cost and scalability issues is the development of automatic systems capable of annotating and extracting relevant content from clinical notes, which has led to greater research efforts in the field of clinical natural language processing (NLP) in the past years. These efforts have led to the creation of international challenges that provide appropriate data sets and enable performance benchmarking of new methods and solutions. The importance of these challenges is widely acknowledged because of the current lack of adequate resources [8], which impedes the development of more advanced solutions [5]. As such, despite the acknowledged interest and value of automated solutions, their development is very complex as it must cope with the challenging nature of working with clinical free text and with the lack of publicly available resources.

Owing to the flexible nature of clinical notes, developed solutions can target the extraction of different types of information from clinical narratives. This process of extracting information is usually split in named entity recognition (NER), named entity normalization (NEN), and relation extraction (RE). NER has the objective of detecting entities of interest in the text, such as diseases or family relatives, whereas NEN is responsible for mapping these entities to normalized concepts in coding standards, such as systematized nomenclature of medicine clinical terms [9] or RxNorm [10] in the case of medical text. RE is focused on detecting relationships between the entities (eg, detecting connections between drugs and adverse drug events) and is very important as it allows the leap from concept extraction to concept understanding [5].

This study focuses on the extraction of the family history component from clinical notes, which can provide insight into disease susceptibility and is important for the prevention, diagnosis, and treatment of specific diseases [11,12]. A demonstration example is the work by Wang et al [13] in which they used a text corpus containing 3 million clinical notes to analyze the patient family history, focusing on family members, medical problems, and their associations, and discovered (1) considerable compliance between positive and negative medical issues mentioned in the reports considering the diagnosis and family history and (2) the existence of medical problems a decade before the diagnosis dates of the determined problem. This study extends a previous contribution [14] by exploring deep learning (DL) approaches for the detection of family history entities in clinical notes and integrating this component in an improved version of the previously developed solution, creating a hybrid system for extracting entities and relations from family history information. The final hybrid solution is provided in a publicly available code repository [15].

The main contributions of this study are as follows:

- This study proposes a strategy to automatically annotate large amounts of EHRs, allowing quick detection of comorbidities with family relations.
- We evaluate the impact of using different DL architectures and embeddings in clinical information extraction.
- We improved the family history information extraction pipeline by combining automatic concept annotations with DL and rule-based architectures to discover entities and relations in the clinical notes.

### Related Work

This study is focused on performing NER on clinical notes to extract family history information, namely, family members and observations such as disease mentions, and on detecting associations between detected entities. Correctly detecting family relatives in clinical notes is far from a straightforward task as the following situations must be considered: (1) notes frequently have cascaded information regarding family relatives (eg, “The patient’s grandmother had cancer in her late 60s [she had a cousin who died from cancer] but his grandfather has no history of cancer.”); (2) notes can mention family members with no blood relations, such as the partners of the patients and their relatives; or (3) the relationship of the family member may not be directly expressed. The existence of such situations where the relationship is complex to understand because of the numerous kinship degrees can eventually lead computational systems to lose context, failing to correctly determine the relationship between the detected entity and the patient. In contrast, disease observations can also be troublesome to detect, as, for instance, they can be mentioned as a sequence of several complex terms or even by disjoint mentions.

Existing solutions typically follow rule-based or machine learning-based approaches; however, it is also possible to combine both approaches in hybrid systems. Furthermore, owing to the reckoned potential of DL approaches in the medical field [16], recent years have shown the emergence of DL-based solutions [5].

For many years, rule-based models were the preferred architecture when developing solutions for extracting family history information, supported by the rationale that, in theory, a good set of rules can manage good concept coverage, thus producing excellent results. Goryachev et al [17] proposed a rule-based algorithm and demonstrated the success of this kind of architecture, whereas Friedlin et al [18] used a rule-based model to extract and code clinical data from clinical reports.

With the growing interest in the development of NLP solutions, generic frameworks such as unstructured information management application [19] and general architecture for text engineering [20] were created to provide support in the development of information extraction systems, from which popular solutions such as clinical text analysis and knowledge extraction system were derived [21]. Despite aiming to offer modular flexible processing workflows that can be reused, these frameworks have the drawback of requiring a deep understanding of the tools given their high-level abstractions.

In contrast with the previous frameworks, toolkits were developed with the goal of providing a set of stand-alone tools that can be easily combined in a processing pipeline. Examples of popular toolkits are the Natural Language Toolkit (NLTK) [22], Apache OpenNLP [23], Stanford CoreNLP [24], and Clinical Language Annotation, Modelling and Processing [25]. Despite the interest in these toolkits, they were developed considering general text instead of biomedical or clinical text, which commonly require specialized tools. Neji was developed to tackle this limitation, providing a modular architecture that integrates specialized modules for biomedical NLP. Thus, it combines the benefits of general frameworks and toolkits with those of specialized tools [26]. These modules can apply different methodologies, such as rule-based models, dictionary matching, and machine learning models. Moreover, Neji provides configurable web services that enable easy integration of its annotation capabilities in external tools [27].

More recently, with the success of DL approaches in text processing problems, DL is being adopted in solutions designed for biomedical and clinical text. One of the key areas where DL has impacted is representation learning, for instance, with the creation of dense representations such as word embeddings. These can be fine-tuned to specific domains and can be easily integrated in other learning algorithms, helping them achieve improved performances in NLP tasks [28]. BioWordVec is an example of publicly available biomedical and clinical word embeddings [29]. However, these embeddings still have the limitation of not considering context, which results in the same word having the same representation when used in completely different contexts (eg, *suits* in *your offer suits our needs* and he *always wears suits*). This was addressed by the development of contextual embeddings such as Embeddings from Language Models [30] and bidirectional encoder representations from transformers (BERT) [31]. These embeddings can also be fine-tuned to specific domains, resulting in the creation of variations such as BioBERT [32] and clinicalBERT [33].

Embeddings are widely used in DL solutions because the resulting dense representations can be easily explored by various DL model architectures. One particular architecture that achieves state-of-the-art results in biomedical and clinical text problems such as NER is the bidirectional long short-term memory (BiLSTM) network coupled with conditional random fields (CRF). Dai et al [34] compared the use of word embeddings (word2vec) and BERT for NER in clinical notes, with a BiLSTM-CRF model, and demonstrated better performance when using BERT to represent clinical text. Li et al [35] used character embeddings, medical dictionaries, and part-of-speech features in a BiLSTM-Att-CRF model, which consists of a BiLSTM with an attention layer bridging the BiLSTM and CRF. This architecture was used to perform clinical NER in EHR notes, and it obtained interesting results, demonstrating the potential of attention mechanisms [35]. More recently, Shi et al [36] used a deep joint learning architecture based on BiLSTMs with word and part-of-speech embeddings for extracting family history information, such as entities and relations from clinical text. Although the demonstrated success of DL approaches at extracting entities and relations from clinical notes, particularly when using BiLSTM-CRF derived architectures, has led to a rapid growth in such solutions, these frequently fail to provide system implementations that hinder their adoption and reproducibility.

## Methods

### Data Set

This work was originally developed under the scope of the 2019 national NLP clinical challenges (n2c2)/open health NLP track on family history extraction, which had the objective of extracting family history information from EHR clinical notes [37]. This challenge track was split into 2 subtasks: the first one being oriented to named entities and the second one focusing on extracting relations between those entities. More detailed descriptions of each subtask are provided in this section. The second subtask directly depended on the first one, as the challenge had the objective of evaluating developed systems as end-to-end family history summarization solutions.

Training and test data sets were provided by challenge organizers. The training data set consisted of 99 unannotated clinical notes, manual annotations of entities and relations for each clinical note, and a gold standard file with eligible entities and relations for the full training set; the test data set consisted of 117 unannotated clinical notes (a gold standard file with eligible entities and relations for the full test set was only provided after the challenge terminated). Both gold standard files contained the annotations for each document without providing any additional information (eg, annotation span or respective line in document). More detailed statistics of data sets are provided in Table 1.

**Table 1:** Detailed data set statistics.

Type		Training	Test	Total
Clinical notes, n (%)		99 (45.8)	117 (54.2)	216 (100)
**Annotated entities, n (%)**				
	Family member	667 (53.4)	583 (46.6)	1250 (100)
	Observation	930 (50.7)	906 (49.4)	1836 (100)
**Annotated relations, n (%)**				
	Family member: living status	376 (51.9)	349 (48.1)	725 (100)
	Family member: observation	740 (49.50)	755 (50.50)	1495 (100)

The first subtask had the objective of identifying family member entities and disease mentions in the clinical notes. When extracting family member entities, it was required to extract both the family relationship (eg, son, father, or uncle) and the family side (eg, maternal). The list of relationships considered was provided by organizers and comprised the following: father, mother, parent, brother, sister, son, daughter, child, grandfather, grandmother, grandparent, cousin, sibling, uncle, and aunt. Any relationship outside the provided list (eg, nephew or great grandparent) should be considered invalid. Moreover, clinical notes could contain family member mentions related to the patient and to the patient’s partner. As the challenge was focused on the patient, all partner-associated family relationships should be discarded.

The second subtask focused on extracting relations between the previously extracted entities and considered 2 types of relations. The first type involved detecting living status mentions, which should be used to assign a living status score to the respective family member entity. This living status score was computed by multiplying the properties of being alive and healthy, where each property could have a value from 0 to 2 (0: no, 1: not applicable, and 2: yes). The second type of relations involved assigning relations between detected disease mentions and the corresponding family members, taking into consideration if the observation was negated or not (eg, nonnegated: *the patient has diabetes* and negated: *there are no reports of cancer*).

### Shortest Dependency Path and Coreference Resolution

**Figure 1:**
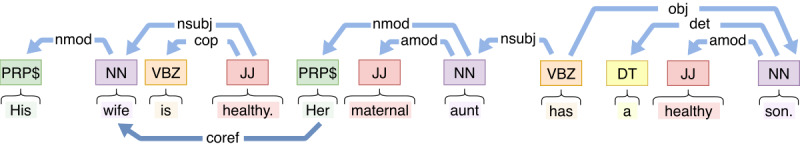
Illustrative example of dependency parsing and coreference resolution from Stanford CoreNLP. amod: adjectival modifier; cop: copula; coref: coreference; det: determiner; DT: determiner; JJ: adjective; nmod: nominal modifier; NN: noun; nsubj: nominal subject, obj: object; PRP$: possessive pronoun; VBZ: verb third person singular present.

The first approach, which was originally used in the challenge submission, combined handcrafted rules and dictionary matching with dependency parsing and coreference resolution. First, a preprocessing step based on Stanford CoreNLP dependency parsing and coreference resolution annotators was applied to all documents. Figure 1 illustrates the result of applying these annotators to an example text fragment.

For the first subtask, the process of entity extraction was divided into 2 subproblems targeting family members and disease mention extraction separately. To extract family member entities, a lexicon was compiled that included all family relationships considered for the challenge, expanded with lexical variants and plural forms, along with others identified by examining an extended family tree, such as partner, great grandmother, nephew, and half-uncle. Although the latter family members should not be considered in the final evaluation, their inclusion was necessary at this stage to avoid erroneous associations with other family members during the following step.

The next step consisted of coreference resolution, for which a coreference graph was created to add the corresponding family member annotations to coreferencing pronouns. Considering the example presented in Figure 1, the family member annotation assigned to the mention *wife* is carried over to the pronoun *her* based on the coreference relation. In the example, this also means that the *maternal aunt* mention gets associated to the *wife* family member. In addition, a process of family relationship resolution was performed by applying a set of rules to map extracted mentions to the corresponding family link, with the resulting family link inheriting the family side if it had been extracted. In the same example sentence, the aunt’s son is mapped to *cousin,* and this carries over the family side mention, leading to the final annotation of (wife’s) *maternal cousin*. Finally, the resulting list of extracted family members was filtered to remove family links other than those targeted in the challenge.

The process of extracting disease mentions consisted of a simpler pipeline, in which a dictionary was first compiled from the unified medical language system Metathesaurus [38]. This dictionary consisted of a filtered version of the Metathesaurus, containing entries only from the Anatomy and Disorders semantic groups, and was used to configure a Neji annotation service. Once the service was set up, all documents were annotated through the web service and a list of extracted mentions per document was created. As this annotation mechanism could introduce many irrelevant entries (false positives) resulting in a lower precision, a false positive list was created by automatically annotating the corpus provided in the SemEval task on Analysis of Clinical Text [39] and identifying false positives against the gold standard annotation. The resulting false positive list was then used to filter the disease mentions extracted in the n2c2 subtask.

For the second subtask, the objective was to extract 2 types of relations for the previously obtained entities. First, a small lexicon regarding living status was extracted from the training corpus, resulting in the following list: *alive, alive and well, dead, deceased, died, doing well, generally healthy, good general health, good health, healthy, living, living and well, otherwise healthy, passed away, stillborn, well, and without problems*. This lexicon was used to extract living status mentions from the documents, which were then mapped into an integer value using the scale previously described in the data set subsection. Finally, the dependency graph created in the first subtask was used to extract the shortest dependency path that associated each disease mention/living status with a family member. This approach disregarded the negation component in observations; therefore, all disease-family member relations were considered nonnegated.

### Rule-Based Engine

The second approach used in the official submissions for the n2c2 challenge track followed a different strategy and consisted of a rule-based engine. This solution involved the creation of rules for family member recognition and dictionaries for observation extraction and processed both subtasks as an end-to-end system outputting the required submission files for both subtasks. After the challenge contribution, this approach was adapted and improved as described further in this section.

The engine processed each sentence in a document sequentially, aiming to link sentences when one of the system processing flows did not detect family members in a sentence. Therefore, using this approach, we created a system that tried to answer the following 3 questions:

1. Who is the subject of the sentence?
2. Which observations are in the sentence?
3. Is the subject alive?

Although answering these 3 questions does not entirely solve the proposed problem, managing to correctly answer them simplifies the process of establishing relations between extracted concepts. The first step in the processing flow splits the document into sentences and removes a considerable set of words. This set was composed of the most common English verbs and the most common conjugations, several adjectives, and names. This procedure preserved relevant words and reduced the distance between words that allowed the correct identification of family members and their respective family side. For instance, for a rule-based system, it is easier to find the family member *cousin* in the cleaned sentence *patient’s uncle son* than in the original sentence *the patients’ uncle has one son*. In this example, this could be erroneously processed as a sentence where the primary subject is the patient’s uncle, instead of the cousin.

After cleaning the sentences, the system applied rules that enable the identification of the subject in the most trivial cases, using exact matching. When no subject was identified, the system processed this using another component, with more complex rules. In this case, rules have more properties such as a set of words that should exist before and after the detected family member, and if this should be discarded or not. These properties enable the generation of very precise rules, which, if used, can increase the potential of the system for the specifications of the challenge at the cost of reducing its reuse in other scenarios (ie, trade-off specificity-generalizing capability).

When no family member was detected with the previous rules, the system executed another component that tried to identify if the sentence currently being processed was related to the previous sentence. If the sentence being processed was the first sentence in the document, the system considered by default that it was related to the patient. Finally, the system ran a last component, which was always executed, to discover whether the sentence was related to the patient or the patient’s partner. If the sentence was associated with the partner, the system discarded the family member entity as required by the challenge guidelines.

Observation extraction consisted of a simpler process than that of family member detection. However, it followed the same principles and used the initial preprocessing for cleaning a set of words. For the challenge, we created a vocabulary based on the observations annotated in the training set and used it in the test set. Simultaneously, the system applied rules to map the detected observation to the identified subject in the sentence. When it was not possible to identify a relation in a sentence, the system did not discard the extracted observations as they were still important for the first subtask.

Living status identification was performed using 2 sets of rules: one targeting deceased subjects and the other targeting healthy and alive subjects. Owing to time constraints, we did not try to identify cases where subjects were alive but not healthy because based on a statistical analysis, mentions for this group of entries represented only 12.2% (46/376) of the living status entries in the gold standard of the training set.

**Figure 2:**
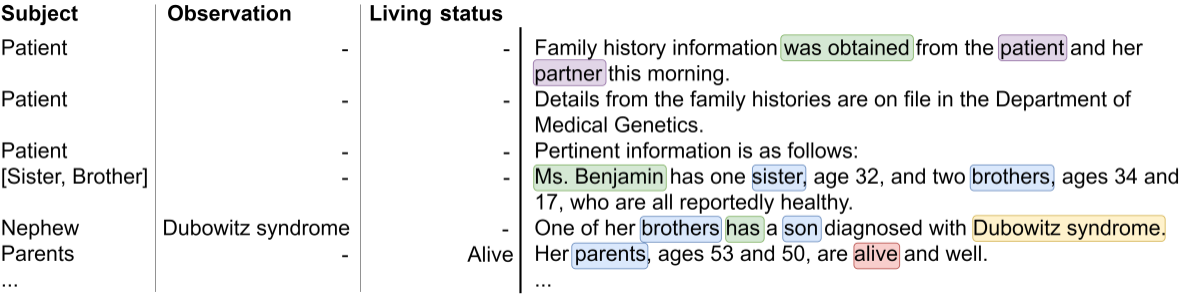
The 3 left concepts represent the main points that the system tries to identify in the text on the right. Highlighted on the right are relevant words for the system to be able to make decisions. Auxiliary words that help identify the subject are represented in green. The words used to identify if the relatives are related to the patient or the partner are highlighted in purple. Blue represents annotated family members, and yellow is used for diseases. Red is used to highlight words concerning subject living status.

The rule-based engine pipeline processes documents individually and sentence by sentence following a sequential flow. In this pipeline, the detected words have different levels of importance. For instance, terms like *partner* and *patient* coexisting in the same sentences are weighted differently. These weights were considered by the complementary rules during subject identification in a sentence. Disambiguation was performed using a set of verbs and specific words in situations where it was not clear whether the sentence was related to the patient, the patient's relatives, the patient’s partner, or the partner's relatives. Figure 2 shows an excerpt of a clinical note that illustrates clearly how the system processes original sentences and what is the result of this processing.

This engine managed good results in the annotation of the family members of the patient. However, the methodology used to extract observations was not the best, regardless of possible improvements to produce more accurate results. Therefore, in a postchallenge contribution, we removed the components for detecting observations and improved components responsible for extracting the family members of the patient and their living status. The living status component was reused with small adjustments to be more generic and compatible with different data sets, yet maintaining the same philosophy of trying only to identify whether the patient is healthy and alive or dead.

**Figure 3:**
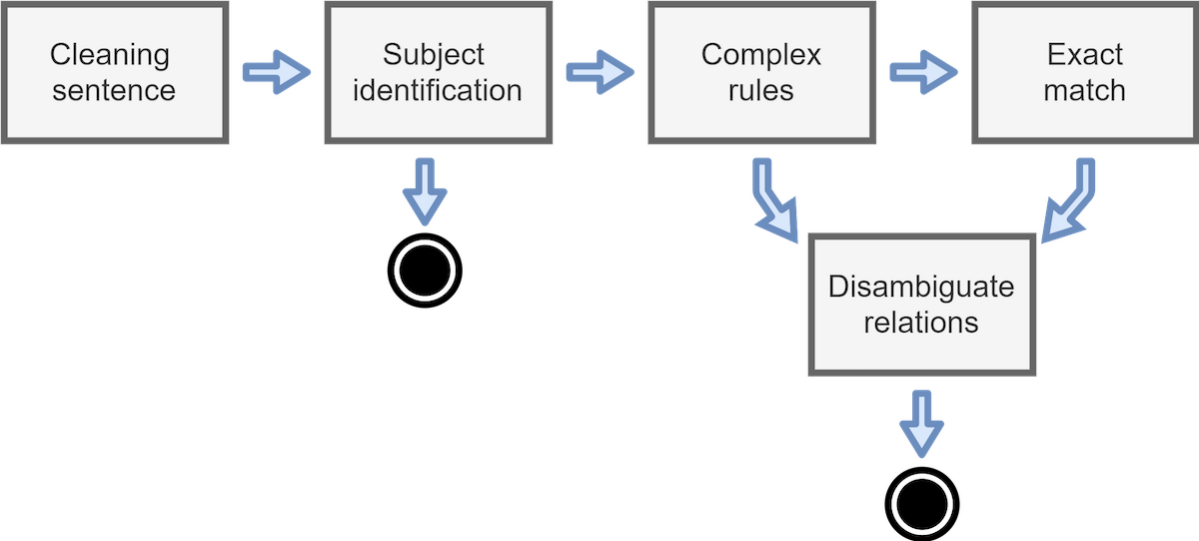
Overview of the processing workflow responsible for family members detection, for the rule-based engine.

The family members annotator was rebuilt following the initial principles but without specific sets of rules that were generated from the training set of the challenge (ie, to reduce overfitting). The system pipeline is presented in a scheme (Figure 3) representing the system pipeline and how components are interconnected. This flow starts by trying to identify if the subject in the sentence is the patient. If not identified, the previously described complex rules are executed. The third component performs exact matching over a clean sentence for trivial annotations, and the output of these components is filtered to disambiguate relations between family members and to remove any relations that should be discarded (eg, to adhere to challenge evaluation guidelines).

In the complex rules component, rules follow a 6-part structure where it is defined the keyword that triggers the rule (eg, *father or grandparent*), and a list of terms that must appear before or after this keyword are defined. Next, this structure contains a flag that indicates whether the annotated relative must be considered or discarded and indicates which is the detected relative. As an example, if the keyword *grandparents* is detected in the clean text, a rule can identify it as a paternal grandparent if there exists the set of words *patients* and *paternal*, in this order, preceding the keyword.

Regarding the disambiguation component, the system contains a set of rules composed of 4 elements. These rules have 2 relatives and a mapping to the real relation of this subject to the patient. As an example, if the component annotates and processes the relatives *father* and *brother*, the system will map them to paternal uncle and return the corrected annotation. Besides the above-mentioned examples, the rule-based system contains a more extensive list of rules that were used for the processes of partial and exact match search.

### DL for Entity Extraction

Owing to the acknowledged potential and success of recent DL solutions in clinical text problems, we extended the original contribution with a novel approach based on DL. The implementation of this solution considered several aspects, namely:

- Following the trend in state-of-the-art solutions, we explored the widely used attention-based BiLSTM-CRF with the attention mechanism placed between the BiLSTM and CRF layers [35] and compared it with a simple linear classifier (with softmax) to evaluate the impact of model architecture in downstream tasks.
- Similar to the approach presented by Yang et al [40], an additional task regarding named entity discovery was integrated with the objective of improving model perception of unknown entities. This downstream task was set as optional; thus, it is possible to train models for NER and for NER and discovery.
- Different types of embeddings were explored for clinical text representation to assess their impact on model performance. BioWordVec word embeddings and clinicalBERT contextual embeddings were used.
- To evaluate the impact of using external resources in model development, Neji annotations were integrated into the input to the model.

**Figure 4:**
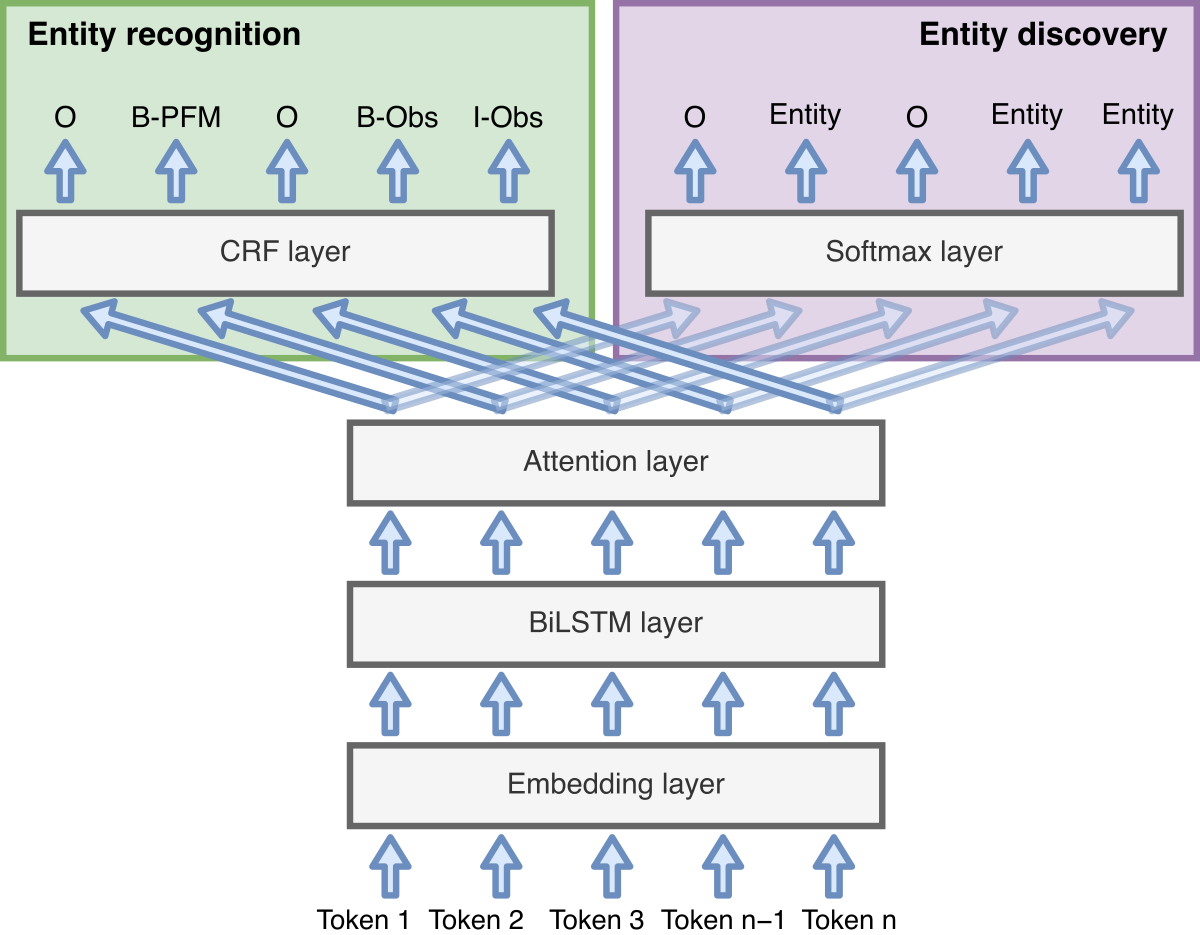
Schematic diagram of the general deep learning model architecture used in this study, showing the 2 possible downstream tasks. The entity recognition task is always executed, whereas the entity discovery task was added as optional to enable model development with and without it. BiLSTM: bidirectional long short term memory; B-Obs: beginning observation; B-PFM: beginning patient family member; CRF: conditional random field; I-Obs: inside observation; n: number of tokens in tokenized sentence; O: outside.

A schematized view of the model architecture used in this study (attention-based BiLSTM-CRF) is presented in Figure 4.

The named entity discovery downstream task consists of a binary classification problem where the system classifies whether an input token is part of an entity or not, disregarding the respective class (ie, if it is an observation or family member mention). This optional task was integrated with the objective of making the model consider the trade-off between discovering more entities and correctly identifying them. When enabled, it is reflected in model training during backpropagation, with the total loss resulting from a linear combination between the losses of both downstream tasks.

Before training any model, it was necessary to preprocess the data set. Text preprocessing began by splitting each document in sentences using the sentence splitter from NLTK, followed by tokenization. However, 2 different tokenization methods had to be used because word and contextual embeddings take different tokenizing approaches: the NLTK word tokenizer was used for word embeddings, and the BERT tokenizer was used for contextual embeddings. The resulting tokenized sentences were tagged using the BIO (beginning, inside, and outside) tagging scheme. Finally, to assess the impact of using external resources, all documents were annotated using Neji, which uses standard vocabularies to detect entity mentions in the input text. Neji annotations, consisting of text spans and entity classes, were then mapped to the tokens in the corresponding sentence, with each token being assigned an integer value similar to the BIO scheme: 0 for tokens not annotated by Neji, 1 for the first token in an annotation, and 2 for the following tokens. The resulting lists of classes were normalized and concatenated to the embedding representations and then forwarded through the BiLSTM layer.

Model training and evaluation were performed using 5-fold cross validation. The Adam optimizer was used, and models were trained with early stopping (the patience parameter can be adjusted). Each training epoch consisted of 100 iterations, during which the training partition was randomly sampled. A detailed list of hyperparameters is provided in Table 2.

**Table 2:** List of hyperparameters used for deep learning model training.

Hyperparameters	Value
Dimension of BioWordVec embeddings	200
Dimension of clinicalBERT^a^ embeddings	768
BiLSTM^b^ hidden size	256
Number of attention heads	2
Epochs	100
Patience	5
Iterations per epoch	100
Dropout rate	0.5
Learning rate	0.001
Batch size	32
Epochs for training BioWordVec embeddings	2

^a^clinicalBERT: clinical bidirectional encoder representations from transformers.

^b^BiLSTM: bidirectional long short-term memory.

In addition, because contextual embeddings provide additional information when compared with word embeddings, we enabled the training of word embeddings for a number of epochs at the beginning of model training, after which the embedding layer was frozen. Finally, as contextual embeddings can partition words in various smaller tokens (eg, *carcinoma* is split in *car,* *##cin,* and *##oma)*, the model could classify only parts of a word as entities (eg, *##cin* and *##oma* classified as entities and *car* as nonentity), resulting in incomplete entities and poor results. Therefore, we added a reconstruction mechanism where the full word is considered when only a part of it is classified as an entity.

The DL approach obtained interesting results in observation extraction but poor performance in family member detection, which goes in contrast with the rule-based approach. As such, we created a final hybrid solution that integrates the DL approach as an observation extraction module in the rule-based engine.

## Results

The original contribution consisted of the development of 2 different approaches for entity and RE: one using shortest dependency paths combined with coreference resolution and another using a rule-based engine. These approaches were validated in the n2c2 challenge on family history extraction. Results obtained in the test data set (Table 3) showed that overall, the first approach performed better in the entity extraction subtask, whereas the rule-based approach performed better in the RE subtask.

**Table 3:** Original overall test results for the 2 national natural language processing clinical challenges subtasks; approach 1: shortest dependency path and coreference resolution and approach 2: rule-based engine.

Subtasks and approach		Precision	Recall	F_1_ score
**Subtask 1**				
	Approach 1	0.6501	0.8892	0.7510
	Approach 2	0.8507	0.6211	0.7180
**Subtask 2**				
	Approach 1	0.5406	0.5005	0.5198
	Approach 2	0.6468	0.5992	0.6221

As the results obtained during the challenge had margins for improvement, and DL-based approaches dominated system submissions in the challenge, we opted to experiment with DL to improve the previous contribution. For the sake of simplicity, tables presenting DL-related results only contain F_1_ score values. However, more detailed results (including precision and recall metrics) are presented in Multimedia Appendix 1.

For the DL-based approach, we started by testing a simple model configuration composed of a linear layer and a softmax function, using contextual embeddings for clinical text representation (Table 4). This simple model served as a reference point to assess the potential of using contextual embeddings to represent clinical text.

**Table 4:** Cross validation results on the training data set (5-fold cross validation) for subtask 1 using a deep learning model composed of clinical bidirectional encoder representations from transformers embeddings, a linear layer, and softmax function, with and without token reconstruction. For simplicity purposes, only F1 scores are presented.

Reconstruction approach and model configuration		Family member	Observations	Overall
**No reconstruction**				
	Baseline	0.3071	0.6620	0.5647
	Baseline+ED^a^	0.1764	0.6397	0.5204
	Baseline+Neji	0.3088	0.7019	0.5924
	Baseline+ED+Neji	0.1840	0.6841	0.5523
**Reconstruction**				
	Baseline	0.3071	0.7444	0.6241
	Baseline+ED	0.1764	0.7142	0.5753
	Baseline+Neji	0.3088	0.7712	0.6418
	Baseline+ED+Neji	0.1840	0.7593	0.6070

^a^ED: entity discovery.

After testing with a simple architecture and evaluating the impact of adding an entity discovery downstream task and external resources to the model, we proceeded to the more complex architecture of the attention-based BiLSTM-CRF, which has been widely explored in the state of the art. This architecture was first tested using contextual embeddings for text representation to assess the impact of model capacity on the resulting model performance (Table 5). After observing the improvements resulting from the change in model architecture, we then evaluated the influence of the embeddings used in the final system results by training the same architecture with word embeddings (Table 5). As word embeddings capture less information than their contextual counterpart, we integrated the possibility of fine-tuning word embeddings for a number of epochs at the beginning of the training process, freezing the embeddings after that point.

**Table 5:** Cross validation results on the training data set (5-fold cross validation) for subtask 1 using the attention-based bidirectional long short-term memory network coupled with conditional random fields with different types of embeddings. When using word embeddings, some configurations enabled embedding fine-tuning for 2 epochs. For simplicity purposes, only F1 scores are presented.

Embeddings type and model configuration		Family member	Observations	Overall
**clinicalBERT^a^**				
	Baseline	0.4103	0.8596	0.7194
	Baseline+ED^b^	0.3788	0.8481	0.7023
	Baseline+Neji	0.3545	0.8478	0.6908
	Baseline+ED+Neji	0.3485	0.8688	0.7081
**BioWordVec**				
	Baseline	0.5921	0.8140	0.7317
	Baseline+ED	0.6553	0.8276	0.7627
	Baseline+ET^c^	0.6166	0.8285	0.7513
	Baseline+ED+ET	0.6219	0.8367	0.7579
	Baseline+ED+Neji	0.7222	0.8529	0.8036
	Baseline+ED+ET+Neji	0.7266	0.8587	0.8092

^a^clinicalBERT: clinical bidirectional encoder representations from transformers.

^b^ED: entity discovery.

^c^ET: embeddings training.

Although the use of a more complex model architecture provided promising results, there was a common trend among all used models, which was the fact that these approaches performed much better at extracting observations than family members.

Considering the fact that the rule-based engine struggled in observation extraction while obtaining good performance in family member extraction [14] and that it performed better in the RE subtask than the shortest dependency path approach, we created a hybrid system that complements the rule-based engine by adding a DL module responsible for extracting disease mentions. Table 6 presents the results obtained with the hybrid solution in the test data set.

**Table 6:** Test results for both subtasks using the final hybrid solution: rule-based engine combined with deep learning module for observation extraction.

Subtask and annotation type		Precision	Recall	F_1_ score
**Subtask 1**				
	Family members	0.7887	0.8307	0.8092
	Observations	0.7523	0.8332	0.7907
	Overall	0.7662	0.8322	0.7979
**Subtask 2**				
	Living status	0.5964	0.6462	0.6248
	Observations	0.4635	0.4371	0.4499
	Overall	0.5100	0.5063	0.5082

## Discussion

### Principal Findings

#### DL for Entity Extraction

Word embeddings have been the go-to method for text representation in the past years. However, contextual embeddings have made a big impact in recent years as they consider positional information and context in the resulting representation, which provides them with higher disambiguation capability than that of word embeddings. As such, our initial tests were performed using publicly available contextual embeddings fine-tuned on biomedical and clinical corpora.

First, we analyzed the impact of reconstructing annotated tokens on the resulting performance. Tests with a simple model (Table 4) showed improved performance in every model configuration when using token reconstruction. However, it is noticeable that only observation extraction benefited from this process, with family member extraction maintaining its F_1_ scores. This is explained by the fact that disease mentions can be very specific and more complex when compared with family members, for instance, the word *mother* is tokenized by the contextual embedding tokenizers as *mother*, whereas *carcinoma* is tokenized as *car, ##cin, and ##oma*. Owing to this different word decomposition, the DL model can classify only parts of the word as an entity, resulting in incomplete entities. The reconstruction procedure solved this issue by adding the missing parts to these entities. Tests with the simple model also demonstrated that the use of external resources such as Neji annotations can help improve entity extraction, whereas adding an additional downstream task regarding entity discovery led to worse results with this model. Finally, it was clear that the model managed to extract disease mentions from clinical notes but failed in the detection of family members, leading to lower overall F_1_ scores.

After performing the initial tests with a simple model and verifying the importance of token reconstruction when using contextual embeddings, we moved to the more complex architecture of the attention-based BiLSTM-CRF (Table 5). To be able to compare it with the previous model, we began by testing the new model with contextual embeddings. Starting with baseline models, it is possible to see that changing to the higher capacity model increased F_1_ scores by approximately 0.1 across all categories. Next, it is possible to observe that complementing the baseline model with the entity discovery task and Neji resources resulted in worse overall F_1_ scores; nonetheless, their combination led to an increase in the F_1_ score for observation extraction (0.8596 to 0.8688).

Finally, to evaluate the influence of using different types of embeddings to represent clinical text, we tested the same model architecture with publicly available word embeddings fine-tuned on biomedical and clinical corpora. Comparing baseline models, word embeddings led to a higher overall performance (0.7317 vs 0.7194), lowering the observation extraction F_1_ score but improving that of family member extraction. Adding extra mechanisms such as external annotations and entity discovery progressively increased model performance, with the final model showing a much higher overall F_1_ score compared with the best contextual embedding configuration (0.8092 vs 0.7194). This higher overall performance was caused by a significant increase in the family member F_1_ score (0.4103 to 0.7266), although observation extraction decreased from 0.8688 to 0.8587 F_1_ score.

The previous results demonstrated that despite the increasing focus on contextual embeddings, word embeddings can obtain good results when using state-of-the-art model architectures. In spite of its much better performance in family member extraction, the word embedding model still obtained subpar performance when compared with the rule-based engine in the same task (0.7266 vs 0.8823). As the objective was to integrate the best approach for observation extraction in the rule-based engine, and contextual embeddings obtained the upper hand in that aspect (0.8688 to 0.8587), we integrated the attention-based BiLSTM-CRF with clinicalBERT embeddings in the hybrid system.

#### Hybrid System

The original rule-based system was developed focusing on the n2c2 challenge and contained sets of rules that were adjusted to the training set. These rules were removed after the challenge, whereas other existing rules were carefully adjusted to create a better system that retained its generalizing capabilities.

With the objective of exploring the best developed approaches for each component of the subtasks, we based the final system on the improved rule-based engine and substituted its weaker component (observation extraction) by a DL-based module. The result was a hybrid system capable of extracting family members and observations along with their respective relations.

As experienced in the original contribution, the results obtained in the test set showed a decrease in performance (Table 6), presenting an overall F_1_ score of 0.7979 in subtask 1 and an overall F_1_ score of 0.5082 in subtask 2. For the first subtask, the hybrid system showed an improvement from the previous best result of 0.7510 overall F_1_ to 0.7979 (a 4.69 percentage point increase). Regarding the RE subtask, although the overall F_1_ score decreased from 0.6221 to 0.5082, there are 2 aspects that should be considered. The first aspect is that adjustments were made to the rule-based engine, which reduced the specificity of its rules and impacted the challenge performance. The second one is that results presented for subtask 2 were obtained using a modified version of the evaluator. The adjusted evaluator performs a more exact analysis of the system output, resulting in lower performance values compared with the original counterpart. A more detailed explanation of this last aspect is provided in the following subsection of *Evaluation and Error Analysis*.

### Evaluation and Error Analysis

The annotations resulting from the approaches described were evaluated using precision, recall, and F_1_ score metrics. The items considered in subtask 1 evaluation were the patient family members combined with their family side and the observations in each document. Regarding family members, if the system does not properly extract relatives’ family side, the results are considered a false positive and a false negative. However, in the case of observations, the evaluator was more flexible. More specifically, if observations were partially annotated (eg, for the observation *diabetes type 2,* the system extracted only *diabetes*), the evaluator considered a true positive. This evaluator was provided by the n2c2 organizers, and we maintained its principles.

The evaluation process for the RE subtask considered (1) the attribution of living status to family members, with correct family side, and (2) the association of observations to family members, including the indication of whether the observation was negated or not. The original evaluator considers each family member, observation, and negation status triple correctly identified by a system. However, the evaluator considers it as a true positive if only the observation or only the negation status were correctly extracted for a given relative. This formulation produces additional true positives, even for annotations that are not completely correct. Therefore, we changed the behavior of this evaluator to consider as true positive only when the system correctly extracted the family member, the respective family side, the (possibly partial) observation, and the observation logical status, as we believe that the extraction is more useful if it is completely correct. As an effect of this change, the F_1_ scores of our challenge submission reduced approximately 10 percentage points when compared with the official results. For instance, when using the new evaluator, approach 1 reduced its F_1_ score from 0.5198 to 0.4431, whereas approach 2 decreased its F_1_ score from 0.6221 to 0.4818.

To understand what affects our results, we randomly selected some false positives and performed a manual analysis on the training set. This analysis led to the detection of inconsistencies in the gold standard annotations, which adversely affected the performance of our system. For instance, in the same clinical notes, 2 identical sentences regarding different family members were annotated with different living statuses. Another example was that at least 14 relatives without living status were annotated when this was present in the gold standard raw data. This raw data consists of the XML files supplied along with the clinical notes in the training set, which were the base of the submission gold standard file. In some of the clinical notes, we detected observations that were present in the text but not annotated in the gold standard and observations that were detected and present in subtask 1 gold standard but not attributed to any subject (despite having the family member also annotated in the gold standard). Although we were not able to perform an in-depth analysis and assess how much this affected our scores, the identified inconsistencies had some impact on performance.

### Limitations and Future Work

The resulting system was built to be more generic than the previous version, which was used in the n2c2 challenge. Despite the improvements made to the system, there are still some limitations. Textbox 1 presents some sentences extracted from the clinical notes that are representative examples of the system limitations.

**Analyses of some of the false positives and false negatives classified by the proposed system. Family member annotations are emphasized in the sentence using italics.**

Child not applicable (N/A)

“Mr. Smith’s father suffers from cancer. He has several *children* through several other women...”

Daughter N/A

“The maternal/paternal great-aunt that has diabetes had several children. One of these individuals had a cancer of an unknown type and is deceased. The second *daughter* is the individual with diabetes type 2...”

Parent N/A

“John’s *parents* are both reportedly healthy at age 63, but they have not seen a physician in approximately 30 years. John’s mother had one second trimester miscarriage...”

Sibling N/A

“Saul’s father is a 39-year-old man who is a college graduate and who has a total of 5 *siblings*...”

Grandparents N/A

“While living in Texas, they lived with extended family, including Peter’s *grandparents*...”

The first example of these limitations concerns the establishment of incorrect sentence connections in certain situations. Depending on the scenario, in the first sentence in Textbox 1, it could be annotated *child* or *sibling*, as it is influenced by the order in which rules are applied during family members detection. However, in this example, the pronoun *he* refers to the patient’s father. Thus, the mentioned children are patient’s half-siblings, a relative that should not be considered according to the guidelines.

The problem in the second example is also related to sentence linking. The system detects a daughter because it loses the sentence context. In addition, the existence of *maternal/paternal* before a relative led to inconsistencies in the detection because there are no rules for these situations. Despite all those problems, the relative annotated as daughter is in fact a third-degree cousin, a relationship that should not be considered. The third and fourth examples show other cases where there was an incorrect family member annotation because of the system losing context within the sentences.

The final example is a special case because the annotation was correctly performed but was not considered in the gold standard annotations, as the clinical notes did not provide any clinical information about the relative. Moreover, the clinical information regarded as necessary for annotating a relative mention is not exclusively composed of observations and may comprehend other types of information such as medication intake or medical procedures, which invalidates the possibility of filtering such situations based on observation associations alone.

Although these might not be the only problems, the limitations presented were those that stood out the most. This led us to analyze possible future work for this contribution, which we could split in different topics. First, we need to test this system in another data set, with a more solid gold standard. This will help us understand the performance of the system as well as its versatility in detail. Another task is the extension of the clinical information extracted. The current version has models designed to extract observations. However, we intend to build other models to extract drugs and procedures, among other medical categories that were not required in the challenge. This extension would lead to a reformulation of the detection of patient’s relatives and allow filtering mentions with no medical information, such as the last example in Textbox 1). Finally, there is also the possibility of exploring machine learning and DL for the process of establishing relations between extracted entities.

### Conclusions

We present an extension to a previous work that focused on extracting family history information from clinical notes. Specifically, we developed a more generic system and improved the previous F_1_ score in the entity extraction subtask by approximately 5 percentage points by combining different approaches. Although the rule-based engine succeeded in extracting patient relatives because of the range of possibilities in the text, this approach failed in the detection of observations. However, the use of DL models helped rectify this gap, with the hybrid system taking advantage of the best characteristics of these 2 methodologies. The hybrid solution is provided in a publicly available code repository.

This study promotes new strategies to easily annotate large amounts of clinical reports currently available in EHR systems. If these reports were annotated and indexed, it would be simpler for a clinician to search for reports mentioning specific concepts. In addition, with data in a structured format, this information can be reused in other scenarios, such as predicting the patient’s susceptibility or predisposition to diseases.

## Appendix

Multimedia Appendix 1 Detailed results for all deep learning model configurations tested in this work. Precision, recall, and F1-scores are provided separately for family member and observation extraction along with overall results.

## Abbreviations

BERT: bidirectional encoder representations from transformers
BiLSTM: bidirectional long short-term memory
BIO: beginning, inside, and outside
CRF: conditional random fields
DL: deep learning
EHR: electronic health record
n2c2: national NLP clinical challenges
NEN: named entity normalization
NER: named entity recognition
NLP: natural language processing
NLTK: Natural Language Toolkit
RE: relation extraction
