# Supplementary material for: Extraction of Family History Information From Clinical Notes: Deep Learning and Heuristics Approach
Source: JMIR Med Inform. 2020 Dec 29;8(12):e22898. doi: 10.2196/22898 (PMC7803476; doi:10.2196/22898)
Supplement: Multimedia Appendix 1 [file medinform_v8i12e22898_app1.pdf]

Model	Configuration	Training Dataset								
		Family Members			Observations			Overall		
		Precision	Recall	F1-Score	Precision	Recall	F1-Score	Precision	Recall	F1-Score
clinicalBERT + linear + softmax (without token reconstruction)	Baseline	0,9179	0,1844	0,3071	0,5874	0,7581	0,662	0,6208	0,5179	0,5647
	Baseline + ED	0,9286	0,0975	0,1764	0,5667	0,7343	0,6397	0,5866	0,4677	0,5204
	Baseline + Neji	0,9118	0,1859	0,3088	0,6326	0,7883	0,7019	0,662	0,5361	0,5924
	Baseline + ED + Neji	0,9444	0,1019	0,184	0,6204	0,7624	0,6841	0,6397	0,4859	0,5523
clinicalBERT + linear + softmax (with token reconstruction)	Baseline	0,9179	0,1844	0,3071	0,6636	0,8477	0,7444	0,6894	0,57	0,6241
	Baseline + ED	0,9286	0,0975	0,1764	0,6348	0,8164	0,7142	0,6511	0,5154	0,5753
	Baseline + Neji	0,9118	0,1859	0,3088	0,6985	0,8607	0,7712	0,7212	0,5782	0,6418
	Baseline + ED + Neji	0,9444	0,1019	0,184	0,6918	0,8413	0,7593	0,707	0,5317	0,607
clinicalBERT + BiLSTM + Att + CRF	Baseline	0,8133	0,2744	0,4103	0,8125	0,9125	0,8596	0,8126	0,6453	0,7194
	Baseline + ED	0,8241	0,2459	0,3788	0,8183	0,8801	0,8481	0,8192	0,6146	0,7023
	Baseline + Neji	0,8162	0,2264	0,3545	0,86	0,8359	0,8478	0,8525	0,5807	0,6908
	Baseline + ED + Neji	0,7926	0,2234	0,3485	0,8419	0,8974	0,8688	0,834	0,6152	0,7081
BioWordVec + BiLSTM + Att + CRF	Baseline	0,9018	0,4408	0,5921	0,9049	0,7397	0,814	0,904	0,6146	0,7317
	Baseline + ED	0,8852	0,5202	0,6553	0,8777	0,7829	0,8276	0,8801	0,6729	0,7627
	Baseline + ET	0,9043	0,4678	0,6166	0,8704	0,7905	0,8285	0,8803	0,6554	0,7513
	Baseline + ED + ET	0,864	0,4858	0,6219	0,8616	0,8132	0,8367	0,8623	0,6761	0,7579
	Baseline + ED + Neji	0,8692	0,6177	0,7222	0,8391	0,8672	0,8529	0,8491	0,7627	0,8036
	Baseline + ED + ET + Neji	0,8947	0,6117	0,7266	0,8483	0,8693	0,8587	0,8633	0,7615	0,8092

ET: Embeddings Training

ED: Entity Discovery

This is a Multimedia Appendix to a full manuscript published in JMIR Medical Informatics.

For full copyright and citation information see http://medinform.jmir.org/2020/12/e22898/
